# Study Protocol for a Randomized Controlled Trial Evaluating the Effectiveness of a Group-Based Self-Determination Enhancement Intervention for Adults with Mild Intellectual Disability and Their Caregivers

**DOI:** 10.3390/ijerph19031763

**Published:** 2022-02-04

**Authors:** Phyllis King Shui Wong

**Affiliations:** Department of Social Work, Chinese University of Hong Kong, Hong Kong, China; pkswong@swk.cuhk.edu.hk

**Keywords:** self-determination, personal well-being, Hong Kong adults with ID, caregivers, randomized controlled trial, group-based intervention

## Abstract

Self-determination is regarded as an adult outcome for people with an intellectual disability (ID). However, self-determination curricula are rarely available in Hong Kong. This paper outlines a protocol for an experimental study that examines the effectiveness of a group-based self-determination enhancement intervention for adults with mild ID and their caregivers. A randomized controlled trial with pre-test, post-test and three-month follow-up is proposed. A total of 120 participants will be randomly assigned to three conditions: self-determination enhancement group, self-determination enhancement PLUS group (with caregivers in a parallel group) and leisure activity group as a control condition. Five groups will be organized for each of the three conditions. There will be 10 sessions per group covering the core components of self-determination including self-knowledge, goal setting and attaining goals, self-regulating and adjusting plans. Components for caregivers include understanding how self-determination and REACH responding skills can support their children to exercise self-determination through positive interaction. Self-determination competencies and personal well-being will be measured at three points in time. The proposed study is the first evidence-based local study aimed at examining a culturally tailored self-determination enhancement intervention for people with ID and fills a research gap in existing interventions. If the intervention is demonstrated to be effective, it will provide new knowledge for a group-based intervention and will be used with Chinese-speaking people with ID in different parts of the world. (Trial registration: ClinicalTrials.gov: NCT05167929)

## 1. Introduction

Personal self-determination was recognized as a human right by the United Nations (UN) in 1967 [1], and Nirje [2] asserts that people with disabilities should be accorded the same right to self-determination as everyone else. The UN Convention on the Rights of People with Disabilities and the World Health Organization have stressed the importance of self-determination (including choice-making, decision-making and participation) for people with disabilities’ dignity and mental health; self-determination is also a key feature and corresponding criterion for the delivery of quality in social services [3,4]. Since the late 1990s, the concept of self-determination has developed significantly in the international field of intellectual disabilities (ID). Many studies have evidenced the importance of self-determination in different areas of the lives of people with ID. Firstly, people with ID who have higher levels of self-determination attain better academic performance [5] and achieve more positive adult outcomes including the following: autonomous community living [6,7]; more independence, being more financially independent or obtaining better job benefits [8,9]; and higher quality of life [10], such as higher life satisfaction, better personal well-being and attaining more meaningful engagement with others [9,11,12,13,14,15]. Self-determination competencies also positively correlate with personal well-being for people with ID in Chinese societies [16], confirming that self-determination is cross-culturally important to people with ID.

Scholars have stated that self-determination is a multifaceted construct [17]. Self-determination theory (SDT) [18,19,20] suggests that self-determination is the capacity to choose and proposes that autonomy, relatedness and competence are three universal and intrinsic human needs that motivate people to become involved in activities [18,21,22]. Autonomy refers to people initiating actions for themselves, from their integrated values and interests and feeling ownership over their actions [19]. The activities might be in accordance with the person’s own thoughts or might be influenced by others [23]. Relatedness refers to a sense of belonging and connection with others; that a person cares for and is cared for by others [19,23,24]. Competence refers to a sense of mastery [19], and a felt sense of confidence [23]. A person experiences satisfaction through overcoming optimal challenges, expressing his or her talents and capacities and feeling able to succeed and grow [19,23]. The functional theory of self-determination suggests that a self-determined person possesses specific capacities (behavioral autonomy and self-regulation) and attitudes (psychological empowerment and self-realization) that coexist in the consistent performance of self-determined actions. The theory also proposes 12 component elements of self-determined behavior (concerning choice-making skills, goal-setting and goal-attainment skills, self-knowledge skills, etc.) [25,26,27]. The theory stresses that one’s capacity for self-determination can be enhanced through learning and can be developed when opportunities to do so are available in one’s environment. The ecological theory of self-determination suggests that self-determination occurs as a result of the ongoing interplay between individuals and multiple environments [28]. An individual is at the center of multiple systems, and his or her self-determination competencies (i.e., attitudes, knowledge and skills) influence how he or she interacts with the external environment with regard to issues of self-determination [29]. The self-determined learning theory stresses that self-determined people are effective regulators in the goal pursuit process; they will try to adjust their expectations or explore more favorable environmental conditions when there is a discrepancy between their existing state and their desired goal [30,31]. Individuals are often in flux between their existing states and personal goals. In the course of the fluidity process, more self-determined people will try to set appropriate expectations when matching their competency (the assessment of their own existing skills, interests and motivations) with the present opportunity; they will take into consideration their own strengths and constraints as well as environmental factors (both human and situational factors) and then make adjustments whenever necessary [32]. The self-determination process involves various elements including self-understanding, personal preferences, goal setting, plan setting, choice making, decision making, problem solving, self-management, plan reviewing and self-regulation. This theory proposes that, to become a more self-determined person, an individual needs to learn to make adjustments when necessary at different stages in the self-determination process.

A social-ecological perspective proposes that family, as a microsystem, plays a crucial role in influencing an individual’s self-determination [33,34]. Studies have shown that the parents of students with ID tend to recognize the importance of self-determination skills; however, they do not engage their children extensively in exercising these skills at home [35]; although parents try helping their children to exercise self-determination skills at home, their approaches differ from what their children learn at school [36,37]. Cultural factors are also regarded as a vital consideration in a person’s development and practice of self-determination [33,38]. The development of self-determination, opportunities to promote it and supporting elements that enable its expression are affected by multiple cultural factors such as race and ethnicity, gender, disability status, socioeconomic status and language [33]. For example, parents from Western cultures pay more attention to independence than those from non-Western cultures [39]. In Chinese collectivist culture, people may be more comfortable with demonstrating their self-determination competencies than their self-determination autonomy [32]. While recent studies conducted in Western countries have begun to stress autonomy support and partnership in decision-making from paid and unpaid caregivers [40,41,42], people with ID in Chinese societies have less autonomy in financial matters and over major life decisions [43].

The literature shows that the effectiveness of self-determination curricula and interventions in enhancing general self-determination skills has been demonstrated. The core components of self-determination curricula and interventions in Western countries mainly comprise self-knowledge, goal-setting and planning skills, choice- and decision-making skills, problem-solving skills, self-advocacy and management skills and self-evaluation and outcome expectation skills. However, these classroom-based self-determination curricula and interventions mainly focus on schooling or transitional periods; while self-determination is relevant across the life span, there is very little curriculum focus on adults [44]. In addition, the majority of self-determination curricula or intervention packages have been developed for students with diverse kinds of disabilities in the school context or at the point of transition to adulthood [31,45,46]. Although the literature has mentioned the importance of working on parents’ values, beliefs and perceptions regarding their children’s potential for self-determination [47] and has identified an ongoing need to provide parents with opportunities to learn about self-determination and how to facilitate their children to exercise it [48], very few studies provide training for parents of people with ID. In recent years, self-determination theory has been applied in diverse fields such as parenting and education, as well as in regard to life goals [49]. Studies in the general population have evidenced the effectiveness of need-supportive [50] and autonomy-supportive practices [51,52] conducted by teachers and parents to enhance children’s and adolescents’ motivation to engage in learning activities and reduce academic stress. Need-supportive practices emphasize taking others’ perspectives and trying to tune in to others’ psychological needs for competence, autonomy and relatedness, as well as demonstrating empathetic responses [50]. Autonomy-supportive practices suggest adopting autonomy-supportive behaviors (e.g., avoiding controlling behavior and language and adopting children’s perspectives) and providing structure (i.e., setting clear goals, expectations and directions) to enable individuals to sustain their perception of competence [51]. These practices also encourage the provision of rationales that foster the internalization of individuals’ motivations [52]. In Hong Kong, there is no well-developed curriculum tailor-made for people with ID, particularly for adults with ID [16]. Therefore, considering the importance of caregivers in self-determination and the cultural characteristics of Chinese societies, the author proposes a group-based, culturally tailored intervention for enhancing self-determination among adults with mild intellectual disability and their caregivers.

### 1.1. Conceptual Framework of the Intervention and Its Delivery Approach

**Figure 1:**
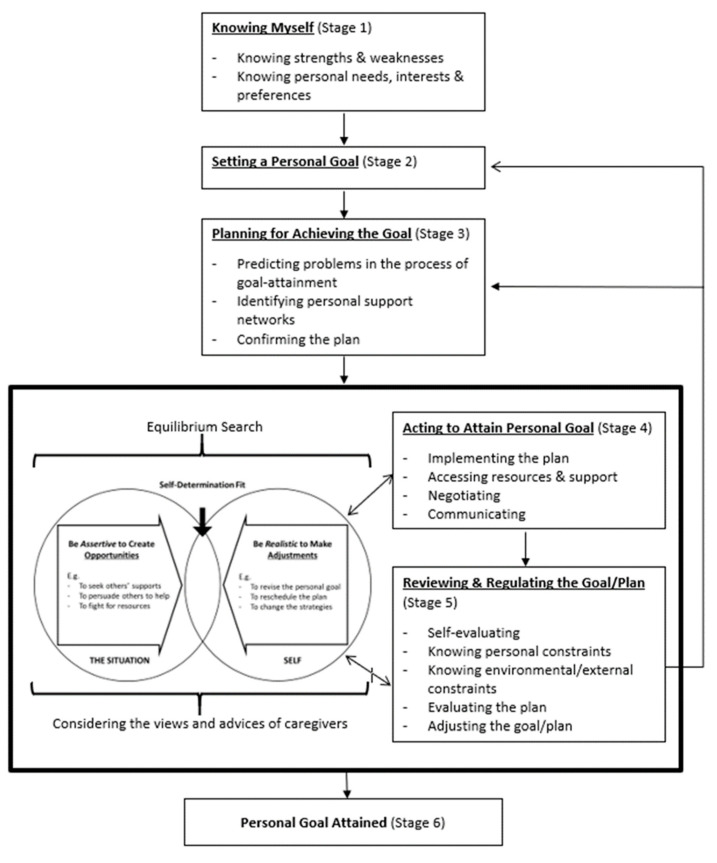
Conceptual Framework of the Group-Based SD Enhancement Intervention.

Studies have recognized that self-determination competencies such as goal setting, planning, choice making and self-regulation are applicable to Chinese people [32,53]. In this regard, the proposed intervention adopts key learning elements from Western self-determination curricula, e.g., [30,46,54], developing a six-stage process of goal pursuit: (1) knowing myself, (2) setting a personal goal, (3) planning for achieving the goal, (4) acting to attain personal goals, (5) reviewing and regulating the goal/plan and (6) personal goal is attained. In stage five, ‘reviewing and regulating the goal/plan’, when there is a need to regulate the goal or the plan, the learners will be facilitated to go back to stage two, ‘setting a personal goal’ or stage three, ‘planning for achieving the goal’ to adjust the goal or the plan accordingly (see Figure 1).

Studies conducted in Hong Kong suggest that family members, particularly parents, play an important role in the exercise of self-determination by people with ID. There is a process of searching for equilibrium in working towards self-determination, in which adults with ID simultaneously consider the views of their parents and concern their feelings when making decisions, while parents struggle between autonomy support and protectiveness, trying to strike a balance in different situations [16,43]. Taking this culturally specific dimension into account, this study’s intervention develops and incorporates the state called self-determination fit at stages four and five; this encourages adults with ID to find the state of self-determination fit during the process of searching for equilibrium (see Figure 1). In the process of acting on the plan, people with ID maintain an ongoing review of themselves and on their situation. When necessary, they should be assertive in creating opportunities for themselves (e.g., seeking support or tangible help from caregivers or others). However, if after recognizing intractable constraints and/or considering caregivers’ or others’ views, they find the plan or the goal is unrealistic, they may then understand that they need to regulate the plan or goal. The process of identifying the state of self-determination fit helps people with ID to clear their minds and recognize their own ways of regulating the plan or goal accordingly.

To enhance caregivers’ autonomy support, based on the aforementioned intrinsic psychological needs and autonomy-supportive practices, this study develops a set of responding skills called REACH (i.e., rationale giving, empathy showing, acting out, companion and highly praising). Providing a rationale using noncontrolling language leads to autonomous motivation [55,56,57,58]. In this intervention, we encourage caregivers to explicitly express a rationale or reasons to the participants, regardless of whether or not they support their children in pursuing the plans (*rationale giving*). Caregivers are also encouraged to show empathy, because the participants may experience ups and downs and may sometimes feel excited and sometimes upset in the course of goal pursuit (*empathy showing*). Besides being psychologically connected, walking together (*companion*) with the participants along the journey of goal pursuit (e.g., rendering practical help or accompanying them to particular activities) is also important as giving these tangible assistance makes things easier than if the participants faced the challenges alone. *Highly praising* includes giving positive feedback and showing the participants recognition and appreciation. These modes of support have the potential to help people with ID become more competent [19] and facilitate them to have more energy and power in pursuing their personal goals. *Acting out* means encouraging caregivers to act out all the above four skills explicitly to show they support the participants in their journey toward goal pursuit.

We propose adopting a group-based mode of delivery for the intervention; the target groups are adults with mild ID and their caregivers. While most empirical self-determination training in Western countries is designed for students and is based on classroom curricula, this study proposes groupwork as the intervention method for adult participants with ID. The advantages of group-based intervention such as modelling, role playing, reciprocal learning, peer support, group problem solving, individual change caused by group dynamics and interpersonal change processes are proven and well-recorded methods [59,60,61]. Therefore, firstly, the groupwork learning environment provides effective moments for participants with ID to learn subjects relating to interactional person-in-environment situations, such as the process of self-determination fit. Secondly, groupwork is more effective than one-way lecturing in helping adult participants integrate the self-determination skills learned in the group into real-life situations. This intervention emphasizes the importance of adults with ID practicing the goal pursuit steps in real-life situations, in accordance with their own individual goals and plans. The participants with ID are thus required to try out their own plans during the intervention (group sessions 6 to 9). Some of their caregivers will be involved in a dyadic intervention, learning what self-determination is and practicing the REACH responding skills to support the participants in their goal pursuit.

The instructional strategies for the intervention recognize that the elements of working memory, a main component of executive function, and emotion regulation have been shown to facilitate people with ID in acquiring skills [62]. In our curriculum, we will employ slogans as well as visual cues such as diagrams, flow charts and videos during training. We anticipate that, through improving their working memory, participants with ID can learn self-determination skills and apply them in their daily lives more easily.

### 1.2. Study Objectives

The objectives of the study are to develop a group-based, culturally tailored self-determination enhancement intervention for adults with ID and to examine its effectiveness.

## 2. Materials and Methods

### 2.1. Study Design, Aims and Hypotheses

**Figure 2:**
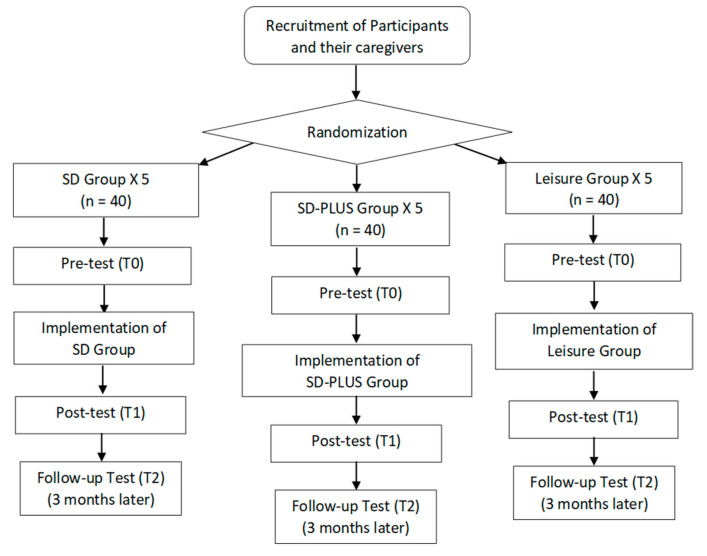
Design Structure of the Study.

This study is a randomized controlled trial (RCT), where adult participants with mild ID and their caregivers are randomized into three arms: (1) self-determination enhancement group (SD group), (2) self-determination enhancement PLUS group (SD-PLUS group) and (3) leisure activity group (comparison group, hereafter called leisure group). SD-PLUS is the only group to include caregivers during the intervention. There will be three assessment points: pre-test (T0), post-test (T1) and follow-up (3 months later) (T2); see Figure 2 for a diagrammatic representation of the study’s design structure. To ensure equity of access to a potentially effective intervention, participants in the leisure group will receive the same SD group intervention following completion of all the assessments for the intervention arm. The study protocol was approved by The Survey and Behavioral Research Ethics Committee of The Chinese University of Hong Kong (reference no.: SBRE-18-234) and the trial was registered in the ClinicalTrials.gov Protocol Registration (registration no.: NCT05167929).

This study aims to evaluate the effectiveness of the intervention. There are three intervention conditions: (1) SD group—only attended by participants with ID; (2) SD-PLUS group—in the form of a parallel group structure, in which the participants’ caregivers will attend the caregivers’ group; and (3) leisure group—only attended by participants with ID. The hypotheses for the study are as follows:

(1) Participants in the SD group and the SD-PLUS group, in comparison with participants in the leisure group, will enhance their self-determination competencies and personal well-being during the post-intervention phase (T1);
(2) Participants in the SD group and the SD-PLUS group, in comparison with participants in the leisure group, will enhance their self-determination competencies and personal well-being at the three-month follow-up (T2);
(3) Participants in the SD-PLUS group, in comparison with participants in the SD group, will exhibit additional enhancements in their self-determination competencies and personal well-being during the post-intervention phase (T1);
(4) Participants in the SD-PLUS group, in comparison with participants in the SD group, will exhibit additional enhancements in their self-determination competencies and personal well-being at the three-month follow-up (T2);
(5) Parent participants in the SD-PLUS group will enhance their beliefs, perceptions and skills in regard to supporting their children in exercising self-determination during the post-intervention phase (T1) and at the three-month follow-up (T2).

It will be a challenge for participants recruitment if three intervention conditions are required. However, since this kind of program has never been tried and tested in Hong Kong, the grant’s vetting reviewers suggested to test the program with three conditions.

### 2.2. Ethical Concerns

Written consent will be sought both from the adult participants with mild ID and their caregivers. People with ID should be accorded as much respect as any other members of the population in terms of their having the autonomy to decide whether or not to participate in research [63]. In addition, this study is concerned with self-determination, and therefore the process of participation is itself a manifestation of self-determination for the participants. Additional measures suggested by the International Handbook of Applied Research in Intellectual Disabilities [64] will be adopted as follows: (1) An individual consent session will be arranged for every potential participant. The content of the consent form will be presented to the participants using plain language and incorporating pictures as visual cues. The participants will then be asked to sign the consent form. (2) A passive consent form will also be used to give dual protection to participants’ rights. Once the participants have signed the consent form, they will then be asked to give consent to us to contact their parents or guardians. Passive consent will then be sought from the parents or guardians.

### 2.3. Sample Size

The sample size is calculated based on a moderate effect size of 0.70 for clinical outcome research. For 80% power, an α error of 0.05, and a test including three independent groups, the required sample size is 105 participants, which is sufficient to conduct Hierarchical Linear Modelling (HLM). Incorporating an estimated drop-out rate of 15% at the six-month follow-up period, the target sample size is 120. Drop-out cases will include participants with ID whose attendance rate in regard to the groups is less than 70%, or those who cannot complete all questionnaires during the three phases. With 6–8 participants per group, 5 SD groups, 5 SD-PLUS groups and 5 leisure groups will be conducted.

### 2.4. Participants

#### 2.4.1. Eligibility

The participants’ inclusion criteria are as follows: (1) Chinese people who have mild ID (i.e., assessed in their most recent psychological report), (2) aged 18 or above, (3) able to master basic comprehension and verbal communication skills and (4) willing to complete the entire intervention. Those who demonstrate severe challenging behaviors will be excluded. Inclusion criteria for caregivers include the following: (1) main family caregivers identified by the participants with mild ID, (2) able to understand Cantonese or Chinese and (3) willing to complete the entire intervention.

#### 2.4.2. Recruitment of Participants

The participants with ID and their caregivers for all arms of the study will be recruited from five non-governmental organizations (NGOs) that provide adult services for people with ID. To achieve diversity in the study samples, the NGOs joining the present study are located in different districts in Hong Kong and currently provide diverse services include community support services, vocational training, employment services and residential services for adults with mild ID. Service users in residential settings have weekly home leaves and they would keep communications and interactions with their families. In addition, service providers reflect that they prefer open recruitment across different settings to recruitment from the same setting. Therefore, no specific service setting is excluded from recruitment. The social workers of the individual organizations will recruit eligible participants and their caregivers based on the inclusion and exclusion criteria for the study. It is known that offering an incentive (e.g., shopping vouchers) to participants upon completion of study is increasingly seen as good practice internationally. However, this study is not able to offer the incentives for the participants since the grant’s budget did not cover this item. Alternatively, the research team is producing additional promotional material (i.e., videos) to facilitate better recruitment. Using lively scenarios and layman language, the videos highlight that participants may benefit from the group intervention itself and their potential contributions to the service development.

### 2.5. Recruitment of Group Facilitators and Co-Workers

The group facilitators and co-workers responsible for the delivery of the group-based SD enhancement intervention are staff members (i.e., registered social workers and direct support workers) of the five participating NGOs. They must have received three sessions of training on the intervention provided by the research team and agree to participate in ongoing group supervision (see Section 2.10 for details).

### 2.6. Randomization

A computerized random number generator in Microsoft Excel is used to allocate participants into the different arms of the study. A research assistant from the research team will undertake the randomization, and the randomization results will be provided to the social workers of the participating NGOs. They are then responsible for contacting the participants, securing their consent and informing them of the session schedule.

Pre-group interviews will be conducted after the randomization. The objective of the pre-group interview is to enhance participants’ understanding of the study’s objectives and the schedule of the intervention groups, and to help group facilitators to familiarize themselves with the learning characteristics and group experience of the individual participants.

### 2.7. Data Collection Schedule

Demographic characteristics and baseline data about the participants will be collected before the group intervention commences (T0). Within one month following the completion of the intervention (T1), participants will be invited to complete the same scales used to collect baseline data (see Section 2.11) to measure any immediate effects. Three months after completion of the intervention, the same study cohort will be invited to complete the scales (T2), so as to measure the sustained intervention effects (see Figure 2). Participants of the SD groups and SD-PLUS groups will also be invited to complete the Self-Determination Action Plan at the end of the intervention. To ensure data quality, the individual face-to-face interviews will be conducted by interviewers who are trained in interviewing skills for people with ID.

### 2.8. Developing and Finalizing the Group Intervention Manual

The Manual of the Group-Based Self-Determination Enhancement Intervention is being developed by the research team, led by the author who is also the principal investigator. The group content is further being developed based on the study conducted in 2006 [65]. The manual includes three main sections: (1) theoretical and conceptual framework of the intervention, (2) principles (e.g., age-appropriateness and real-life learning) and instructional strategies (e.g., picture-based task analysis, flowchart, video-playing, role-playing and small group discussion) of the intervention and (3) the session plans (each session plan includes the objectives of the session, the rundown and time allocation, the detailed content of each section, remarks and practical tips for the group facilitator).

An expert panel will be formed to build the fidelity of structure. The panel will comprise three adults with mild ID, two caregivers of people with ID and two social workers who have more than 10 years’ experience working with people with ID. The panel members are invited to review the framework, content and specific instructional materials for the intervention. A user-friendly version is prepared for the members who have ID and they are invited to attend a practice session in which they try out the activities and exercises of the key sections. All members’ comments and views will then be collected and considered. The group manual will then be provisionally completed for the pilot study.

A trial intervention group will be conducted with eight group members, who meet the same inclusion and exclusion criteria of the later full-scale research study, and their parents. During this pilot study, the research team will test the intervention hardware (e.g., content accuracy, the effect of the multiple instructional strategies, time scheduling, etc.). The team will also accumulate soft skills to manage the software (e.g., ice-breaking and rapport-building skills, skills to manage group dynamics, skills to facilitate and motivate members’ learning, etc.). These soft skills can be transformed into fidelity to the process. The group manual will be further fine-tuned and then finalized according to the experience collected during the pilot study.

### 2.9. Intervention

#### 2.9.1. SD Group

The participants with ID allocated to the SD group will receive 10 weekly, 1.5-hour group sessions. Table 1 provides an overview of the two group interventions. Most sessions will start with a review of the home assignments and key content of the previous session (except session 1). The participants will learn new skills, using a case discussion, and then practice applying the new skills in their own situations. Home assignments will be assigned at the end of each session (except session 10); this will facilitate the participants to act on their own plans using their newly acquired knowledge and skills. There will be a two-week interval between session 6 and 7, to give the participants longer to implement their plans.

#### 2.9.2. SD-PLUS Group

The elements of REACH will be incorporated into the process for this group. Those participants with ID allocated to the SD-PLUS group will receive the same group content as those in the SD group, with the exception of session 8. This session will be renamed session 8+ for this group and will be a joint session with the participants with ID and their caregivers. The caregiver participants will be required to attend a four-session parallel group. The first two sessions are about what self-determination is and its importance, and using the REACH skills to support their children in implementing their goal pursuit plans. In session 3 (i.e., the joint session, which is also session 8+ of their children’s group), the caregiver participants will observe the performance of their children during the session and do dyadic exercises with them. After the joint session, the caregiver participants will attend the last session to review and share their experience and reflections in the joint session, and consolidate what they learned in the group. These four sessions are conducted bi-weekly, and each session lasts for 1.5 h (except the third session that lasts for 2 h) (see Table 1).

#### 2.9.3. Leisure Group

The participants with ID in this comparison group will attend 10 sessions of social activities that are unrelated to the SD enhancement model, with each session lasting for 1.5 h. For ethical reasons, the participants will be invited to receive the same content delivered in the same format as the SD group after the study is completed. This will be conditional on the SD group leading to significant enhancement on the primary outcome. If the SD-PLUS group proves to be effective, the participants will then receive the same content delivered in the same format as the SD-PLUS group. However, since the grant budget item does not cover this stage of data collection, the data would not be analyzed and reported.

### 2.10. Fidelity Check

The fidelity for the implementation of the intervention will monitored by three forms of measurement—for context, compliance and competence fidelity [66]. Context fidelity describes the necessary precursors to high-level performance. To ensure high context fidelity, all groups will be run by facilitators who are registered social workers with at least five years of experience working with people with ID. Their co-workers should also be registered social workers who have at least two years of experience working with people with ID, or direct support workers who have at least five years of experience. Before the start of the groups, all facilitators and co-workers will be required to attend a three-session training course conducted by the principal investigator and the research team to familiarize themselves with the content of the group manual and to learn specific skills for running the groups.

Compliance fidelity focuses on ensuring that the core intervention components are clearly described. To achieve this, all group facilitators and co-workers will be provided with detailed information, work sheets and props to be used in the group sessions. All group sessions will be videotaped. Regular group supervisions will be conducted by the principal investigator and the research team. During supervision, selected video episodes from the group sessions will be played back and discussed. Any difficulties encountered by the group facilitators and co-workers will be discussed, and it is expected that alternatives and solutions will be generated. The supervision sessions are also expected to lead to practice reflections and the accumulation of good practice methods among the facilitators and co-workers, through a mutual learning and support processes.

Competence fidelity measures how well the facilitators are performing the core intervention components of the evidence-based practice. A fidelity checklist is developed that covers the assessment of these areas: (1) instruction on the core self-determination components, (2) enabling the participants to participate and (3) facilitation of interactions among participants. Five experienced social workers who have worked with people with ID for more than 10 years, and who have no affiliation with this study, will be the fidelity reviewers. Video clips of three sessions from each group will be randomly selected, watched and rated by two reviewers who are randomly paired to achieve inter-rater reliability. Each reviewer will review 12 sessions from four groups.

### 2.11. Outcome Measures

A multiple method of data collection will be used to examine both the statistical and clinical significance level of the intervention and the effectiveness of the intervention.

#### 2.11.1. Primary Outcome

The primary outcome is self-determination of participants with ID. Participants with ID will complete the AIR Self-Determination Scale: The Chinese Version (AIR SDS-C) [32] that is composed of two parts. Part one is a 24-item, 5-point Likert-scale questionnaire to measure self-determination in people with ID, consisting of scores from four domains including self-determination competencies, feelings, school and workplace opportunities and home or hostel opportunities. Higher scores denote higher levels of SD competency. Confirmatory factor analysis (CFA) has revealed a very similar factor structure to the original English version; all the factor loadings were between 0.42 and 0.76, and all items were thus retained. The results of the CFA suggested a relatively good fit to the data (χ2(df = 247, *n* =356) = 392.00, χ2/df = 1.59, RMSEA = 0.041, 90% CI [0.033–0.048], CFI = 0.933, TLI = 0.926, SRMR = 0.05). The Cronbach’s alpha (α) value for the entire scale was 0.88 [32].

#### 2.11.2. Secondary Outcome

The secondary outcome is the personal well-being of participants with ID. Participants with ID will complete the Personal Well-Being: Intellectual Disability (Cantonese) 3rd Edition (PWI-C) that was translated from the Personal Well-being Index developed by Cummins in 1996; Cummins and Lau validated the Cantonese version [67]. The index uses an 8-item, 5-point Likert-scale questionnaire to measure the subjective well-being of people with ID; higher scores indicate better personal well-being. The Kaiser–Meyer–Olkin (KMO) values were >0.80 and the Bartlett’s test also reached statistical significance (*p* < 0.05); the Cronbach α coefficients were 0.80 [68].

#### 2.11.3. Process Outcome

A Self-Determination Action Plan will be developed by the principal investigator and research team. The measure of its effectiveness, using the vignettes method, will examine to what extent the participants with ID in the intervention group can transfer their learning into daily life. The participants will be given several scenarios and will then be required to say what their personal goal is and how they will achieve it; for example, questions such as “give an example of a goal you are working on” or “what are you doing to reach this goal?” will be asked. Self-determination skills, including goal setting, choice making, problem solving and decision making, will be tested. A marking scheme will be developed to quantify the behavioral changes in the participants.

#### 2.11.4. Parents’ Outcomes

A questionnaire will be developed by the principal investigator and research team to measure the perceived changes of the parent participants in the SD-PLUS group in regard to their beliefs, perceptions and skills related to self-determination after the intervention. The questionnaire consists of five items measured on a 5-point Likert-scale. For example, questions such as “I do more agree that self-determination is important to my child with ID” and “I grasped the REACH skills and apply them to support the self-determination of my child with ID” will be asked.

### 2.12. Statistical Analysis

Analyses of the outcomes will be performed using the intention-to-treat principle; this method is used to avoid the effects of crossover and drop-out in clinical research. Parameters for those who withdraw will be treated using maximum likelihood estimation (i.e., the full information maximum likelihood method). This method produces approximately unbiased results across a variety of parameter estimates, particularly with a small sample size [69]. Prior to all hypotheses testing, this study will conduct a Chi-square test and a *t* test to assess whether or not the sociodemographic characteristics and the baseline conditions of the participants with ID are different among the groups. Analyses of treatment outcomes will be conducted using hierarchical linear modelling (HLM), which is suggested to have more flexible data requirements (dealing with the missing data), and stresses individual change over group differences. Moreover, the HLM analysis is able to identify differences between conditions through the presence of a significant time x treatment interaction term. To examine the magnitude of change in the three groups at post-test and follow-up, effect size statistics will be calculated using Cohen’s d [70]. Finally, regression analyses will be performed to examine the relationships between changes in self-determination competencies and personal well-being among the participants with ID.

## 3. Discussion

Developing effective interventions to promote the self-determination of people with ID has been a priority for intellectual disability services. Self-determination curricula have been widely developed and implemented in the Western context. However, their application and effectiveness in non-Western contexts have been under-developed and have not been well-studied. The current study aims to develop a systematic curriculum with a group-based intervention to address gaps in both research and practice. This protocol describes a RCT with comparison group design to test whether the proposed group-based intervention exerts a positive effect not only on the self-determination competencies of people with ID, but also on their personal well-being.

This protocol has three key strengths. First, to the best of our knowledge, it will be the first study to develop and examine a systematic self-determination intervention for adults with ID in Chinese societies. Second, the intervention is in a group format that facilitates effective social learning and mutual support, which has received less attention in the ID field. Lastly, the intervention involves caregivers, who help to develop the dyadic concept of interaction around self-determination.

Successful confirmation of the effectiveness of the intervention may have contributions and implications for theory, research and practice. The current study will provide new knowledge on self-determination effects, as well as on personal well-being, by evaluating the effectiveness of a self-determination enhancement intervention model, delivered in group settings, for adults with ID and their caregivers. As a result of the culturally tailored elements of the intervention, the findings will also generate new knowledge about self-determination enhancement methods applicable to adults with ID in the Chinese context. This will significantly extend the existing literature in these areas.

If proved to be effective this intervention model can provide rigorous, structured and evidence-based guidelines for self-determination enhancement practices in Hong Kong and other countries. There are concrete session plans and protocols for the group facilitators to follow. Specific knowledge and skills (e.g., goal-setting DIY, problem-solving funnel, 4-W tool, diagram of my personal and/or external constraints, identifying self-determination fit, on my feasible way and REACH skills for caregivers) are also imparted during the intervention. Both disability practitioners and service users with ID and their caregivers will benefit from the study. Besides the contribution to the development of a reciprocal mode of self-determination intervention in the adult services field in Hong Kong, it may have a further impact on local special education. The protocol has the potential to serve as a useful reference for special educators, pointing them towards better ways of facilitating adolescent students with ID in developing their plans for transition to adulthood and to independent community living.

Further studies in this area should consider a longer follow-up period to evaluate the sustainability of the intervention’s effects. In addition, we suggest it would be beneficial to conduct further studies on strengthening the dyadic interaction between people with ID and their caregivers, as well as on the triangular interaction (involving disability professionals) in achieving enhancements in self-determination.

## Figures and Tables

**Table 1:** Session Theme and Content.

Session	Theme	Content
SD Group and SD-PLUS Group—Participants with ID		
1	Self-knowledge	- To understand own strengths and weaknesses, personal needs, interests and preferences
2	Goal setting	- To learn the ‘5-Step Dream Pursuit’ - To learn the ‘Goal-Setting DIY’
3	Predicting difficulties and identifying personal supportive networks	- Video case illustration - Small group discussion - To complete ‘My Resources and Networks’
4	Problem-solving skills	- To learn the ‘Problem-solving Funnel’
5	Planning for goal attainment	- Video case illustration - To apply the ‘4-W Tool’ to drafting the plan - To complete the ‘Action Plan for Achieving the Goal’
6	Accessing resources and support	- Video case illustration - Role play - To practice seeking support from others assertively
7	Knowing constraints	- To review the progress of own plan implementation - Video case illustration - To be aware of internal/external constraints - To complete the ‘Diagram of My Personal and/or External Constraints’
8	Tackling the not easy to be removed constraints	- Video case illustration - To review own situations and to understand some constraints are not easy to overcome at the moment - To come up with the state of self-determination fit - To find out own feasible ways to achieve the goal through the exercise ‘On My Feasible Way’
8+	Tackling the not easy to be removed constraints and practicing REACH	- Video case illustration - To review own situation and to understand some constraints are not easy to overcome at the moment - To come up with the state of self-determination fit - To find out own feasible ways to achieve goal through the exercise ‘On My Feasible Way’ - Caregiver participants to practice the REACH skills
9	Reviewing the plan/goal	- Video case illustration - To regulate the goal/plan if necessary
10	Round-up	- To consolidate their learning - To reflect their gains
SD-PLUS Group—Caregiver Participants		
1	What is self-determination	- To understand what self-determination is and how is it important to people with ID
2	REACH skills	- To learn what the REACH skills are and how to apply them in daily interactions with their children
3	Same as Session 8+	- Same as Session 8+
4	Round-up	- To review and reflect their observations and discoveries from the Session 8+ - To consolidate their learning

## Data Availability

The data presented in this study after completion are available on request from the corresponding author.
